# Association of circulating miRNAS in patients with Alstrőm and Bardet-Biedl syndromes with clinical course parameters

**DOI:** 10.3389/fendo.2022.1057056

**Published:** 2022-11-25

**Authors:** Agnieszka Zmyslowska, Urszula Smyczynska, Marcin Stanczak, Krzysztof Jeziorny, Agnieszka Szadkowska, Wojciech Fendler, Maciej Borowiec

**Affiliations:** ^1^ Department of Clinical Genetics, Medical University of Lodz, Lodz, Poland; ^2^ Department of Biostatistics and Translational Medicine, Medical University of Lodz, Lodz, Poland; ^3^ Department of Pediatrics, Diabetology, Endocrinology and Nephrology, Medical University of Lodz, Lodz, Poland; ^4^ Department of Radiation Oncology, Dana-Farber Cancer Institute, Boston, MA, United States

**Keywords:** Alstrom syndrome, Bardet-Biedl syndrome, obesity, type 2 diabetes, insulin resistance, lipids, miRNA - microRNA

## Abstract

**Background:**

Patients with the rare syndromic forms of monogenic diabetes: Alström syndrome (ALMS) and Bardet-Biedl syndrome (BBS) have multiple metabolic abnormalities, including early-onset obesity, insulin resistance, lipid disorders and type 2 diabetes mellitus. The aim of this study was to determine if the expression of circulating miRNAs in patients with ALMS and BBS differs from that in healthy and obese individuals and determine if miRNA levels correlate with metabolic tests, BMI-SDS and patient age.

**Methods:**

We quantified miRNA expression (Qiagen, Germany) in four groups of patients: with ALMS (n=13), with BBS (n=7), patients with obesity (n=19) and controls (n=23). Clinical parameters including lipids profile, serum creatinine, cystatin C, fasting glucose, insulin and C-peptide levels, HbA1c values and insulin resistance (HOMA-IR) were assessed in patients with ALMS and BBS.

**Results:**

We observed multiple up- or downregulated miRNAs in both ALMS and BBS patients compared to obese patients and controls, but only 1 miRNA (miR-301a-3p) differed significantly and in the same direction in ALMS and BBS relative to the other groups. Similarly, 1 miRNA (miR-92b-3p) was dysregulated in the opposite directions in ALMS and BBS patients, but diverged from 2 other groups. We found eight miRNAs (miR-30a-5p, miR-92b-3p, miR-99a-5p, miR-122-5p, miR-192-5p, miR-193a-5p, miR-199a-3p and miR-205-5p) that significantly correlated with at least of the analyzed clinical variables representing an association with the course of the diseases.

**Conclusions:**

Our results show for the first time that serum miRNAs can be used as available indicators of disease course in patients with ALMS and BBS syndromes.

## Introduction

Alström syndrome (ALMS) and Bardet-Biedl syndrome (BBS) are progressive and complex genetic syndromes belonging to syndromic forms of monogenic diabetes, the essence of which is the presence of obesity associated with many other pathologies, such as type 2 diabetes mellitus (T2DM), progressive visual and hearing impairment leading to vision loss and deafness, neurological, renal, hepatic, endocrine, pulmonary and cardiac disorders (1). These syndromes are examples of ciliopathies, a group of diseases determined by abnormalities of the primary cilia present in many organs, whose functions are impaired in ALMS and BBS syndromes (2, 3). This results in highly variable clinical manifestations in patients with both syndromes.

The first symptom of ALMS is obesity already present in infants, accompanied by insulin resistance and T2DM in most patients between 14 and 20 years of age (4, 5). In addition, patients show progressive retinal degeneration, hearing impairment, short stature, dilated cardiomyopathy in about 60% of patients, bronchial asthma and pulmonary symptoms (50%), chronic hepatitis with steatosis (50%), chronic renal failure, hypothyroidism, scoliosis, lipid abnormalities, urological and neurological disorders and school difficulties (6–8). All these disorders are determined by autosomal, recessively inherited pathogenic variants in the *ALMS1* gene (9, 10). The symptoms in patients with BBS are very similar to ALMS with the addition of limb bone abnormalities, manifesting mainly in the form of polydactyly, brachydactyly and syndactyly. Patients with BBS syndrome also show cognitive impairment, intellectual disability, renal and cardiac defects and liver fibrosis (11–13). The phenotype of patients is due to causative variants located in multiple genes of the BBSome complex and inherited predominantly autosomal recessively (14, 15). To date, there are no defined markers of disease progression and causal treatment in both syndromes.

MicroRNAs (miRNAs) are small non-coding ribonucleic acid molecules that regulate many important biological processes through RNA silencing and post-transcriptional regulation of gene expression. Specifically, circulating miRNAs are a promising group of biomarkers that have been used as diagnostic and prognostic indicators in obesity, insulin resistance and T2DM (16, 17).

The aim of this study was to determine the profile of circulating serum miRNAs in patients with ALMS and BBS, compare the levels of miRNAs to those in obese patients and healthy subjects and evaluate the associations between miRNA expression and observed metabolic disorders.

## Material and methods

### Patients

The study protocol was approved by the University Bioethics Committee at the Medical University in Lodz, Poland (RNN/343/17/KE). Either the patients or their parents gave their written informed consent for participation in the study.

All 13 patients with ALMS (M/F: 9/4 belonging to eight families) and 7 with BBS (M/F: 5/2 belonging to seven families) had biallelic mutations in the respective genes such as *BBS6*, *BBS8*, *BBS9* and *BBS10*, as confirmed by direct sequencing of the *ALMS* and *BBS* genes, as described previously (18–20), and clinical symptoms indicative of the respective syndrome. Moreover, a group of 19 obese patients and a group of 23 healthy controls were recruited. Healthy controls were selected as individuals without overweight/obesity, glucose metabolism disorders or chronic diseases. BMI-SDS was calculated using population data from Palczewska & Niedźwiecka centile charts (21), after log-transform of raw data as BMI typically have log-normal distribution.

In addition, patients in the study group (with ALMS and BBS) had their clinical parameters evaluated by routine laboratory methods, including: lipids profile, kidney function parameters – serum creatinine and cystatin C levels, metabolic parameters - fasting glucose, insulin and C-peptide levels, HbA1c value and insulin resistance based on HOMA-IR–homeostatic model of insulin resistance assessment (detailed clinical characteristics in **Table 1**).

**Table 1:** Clinical characteristics of the patients from the study groups.

Parameter	LMS patients Median (IQR)	BBS patients Median (IQR)	Obese patients Median (IQR)	Controls Median (IQR)
Age (years)	18.9 (15.8, 22.9)	10.7 (7.2, 16.3)	14.6 (10.9, 17.1)	16.0 (10.3, 17.9)
BMI (kg/m^2^)	30.5 (27.4, 34.9)	29.9 (25.9, 30.6)	31.4 (29.0, 34.2)	20.3 (16.9, 21.5)
BMI SDS	4.2 (3.2, 4.7)	3.7 (3.4, 4.2)	4.1 (3.4, 4.6)	0.1 (-0.3, 0.5)
Total cholesterol (mg/dL)	174.0 (168.0, 193.0)	171.0 (162.0, 219.5)	N/A	N/A
HDL-cholesterol (mg/dL)	38.0 (29.5, 44.0)	43.0 (38.0, 47.5)	N/A	N/A
LDL-cholesterol (mg/dL)	121.0 (102.0, 140.5)	168.0 (116.5, 173.5)	N/A	N/A
Triglycerides (mg/dL)	139.0 (104.5, 309.0)	157.0 (122.0, 177.8)	N/A	N/A
Creatinine (mg/dL)	0.87 (0.76, 0.95)	0.83 (0.78, 0.95)	N/A	N/A
Cystatin C (mg/dL)	0.90 (0.83, 1.11)	1.20 (0.82, 1.44)	N/A	N/A
Glucose (mg/dL)	88.5 (7.0, 107.8)	80.0 (71.0, 85.0)	N/A	N/A
Insulin (IU/l)	27.0 (14.3, 33.4)	7.5 (5.1, 10.3)	N/A	N/A
HOMA-IR	5.9 (3.2, 9.1)	1.6 (1.3, 2.0)	N/A	N/A
Hba1c (%)	5.7 (5.6, 6.9)	5.3 (5.3, 5.9)	N/A	N/A
C-peptide (ng/mL)	6.2 (3.1, 7.7)	1.9 (1.2, 2.0)	N/A	N/A

Continuous data are presented as medians with values of the lower and upper quartile (IQR). ALMS, Alström syndrome; BBS, Bardet-Biedl syndrome; BMI, body mass index; HOMA-IR, homeostatic model of insulin resistance assessment; N/A, not available.

Quantification of miRNA expression was performed in all groups in 2 ml of serum collected during routine testing at their medical centres.

### Analysis of miRNAs

Quantitative PCR miRNome Human panels I&II (Qiagen, Hilden, Germany) were used to profile miRNAs present in the human serum. miRNAs detectable at Ct<37 in at least half of samples in each of the four groups were used for the analysis.

### Statistical analysis

Potential batch effect in results of miRNA expression assay was evaluated using principal component analysis (PCA) and was removed using ComBat algorithm (22). Then, miRNA expression was normalized to the mean expression of all miRNAs detectable in all samples (n=80). miRNA expression values were compared between groups using limma with group (ALMS-, BBS-, obese patients or controls) included in the model as the only candidate variable explaining differential expression (23). The relationships between the results of the clinical course parameters and miRNA levels were assessed using Spearman correlation test. Hierarchical clustering was performed using miRNAs dysregulated in BBS or ALMS *vs.* at least 2 other groups (p<0.05), with Euclidean distance as distance measure between samples and (1 – correlation coefficient) as distance measure for miRNAs, and Ward linkage algorithm. PCA was performed using all miRNAs that passed filtration criteria with missing data imputed with mean expression of miRNA across all samples. Otherwise than for PCA missing value imputation was not used in this study. Due to the low statistical power resulting from small number of patients, no multiple comparisons correction was applied and p values lower than 0.05 were considered statistically significant. For limma results an additional significance criterium was absolute value of log2-transformed fold-change (FC) greater than 0.5 (equivalent to FC>1.41 or FC<0.71).

Statistical analyses were performed with Python 3.8 (including PCA from scikit-learn package, correlation analysis and hierarchical clustering from SciPy) and R 3.6.3 (limma). Graphs were created using Python packages: Matplotlib and Seaborn. If not specified otherwise, we used -ΔC_t_ values in analysis and plots.

## Results

**Figure 1:**
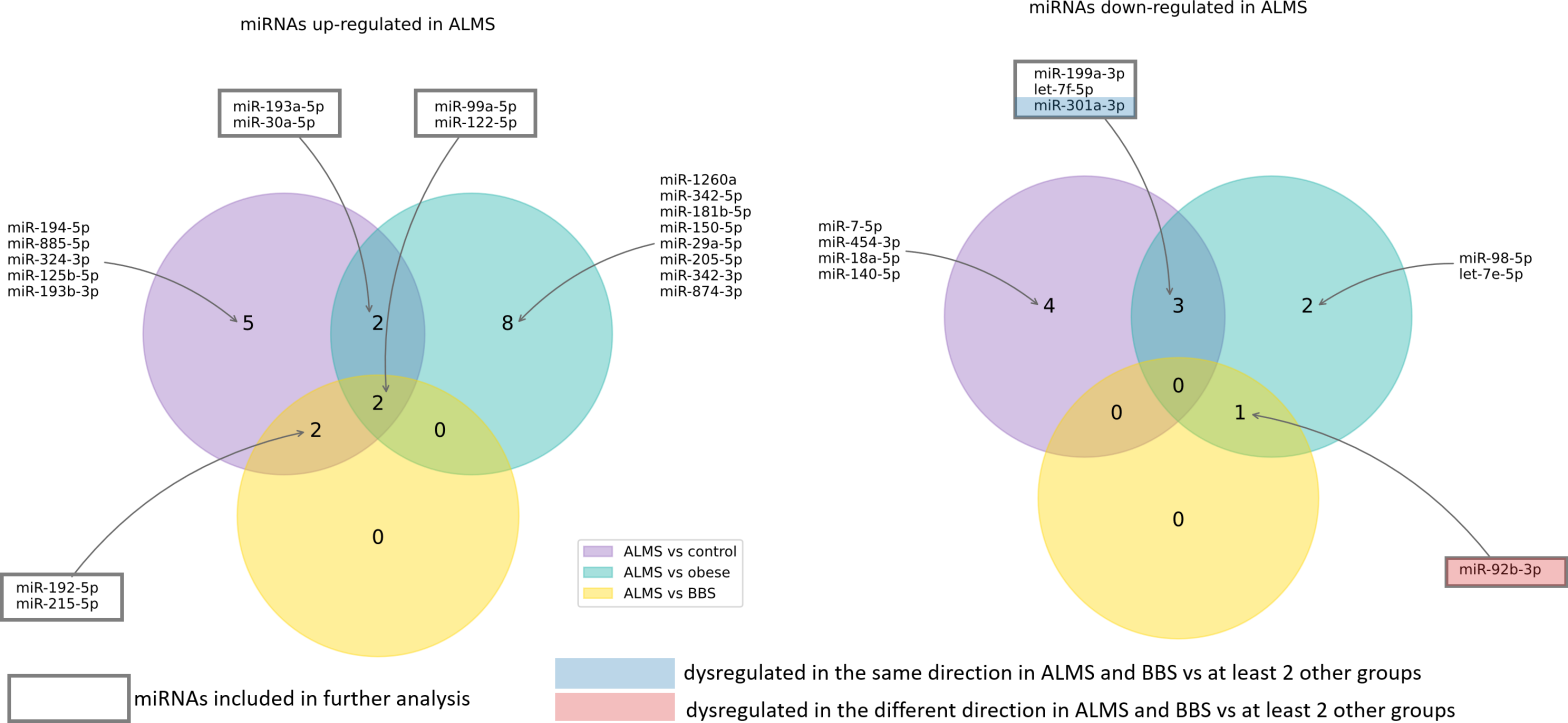
MiRNAs dysregulated in ALMS patients.

**Figure 2:**
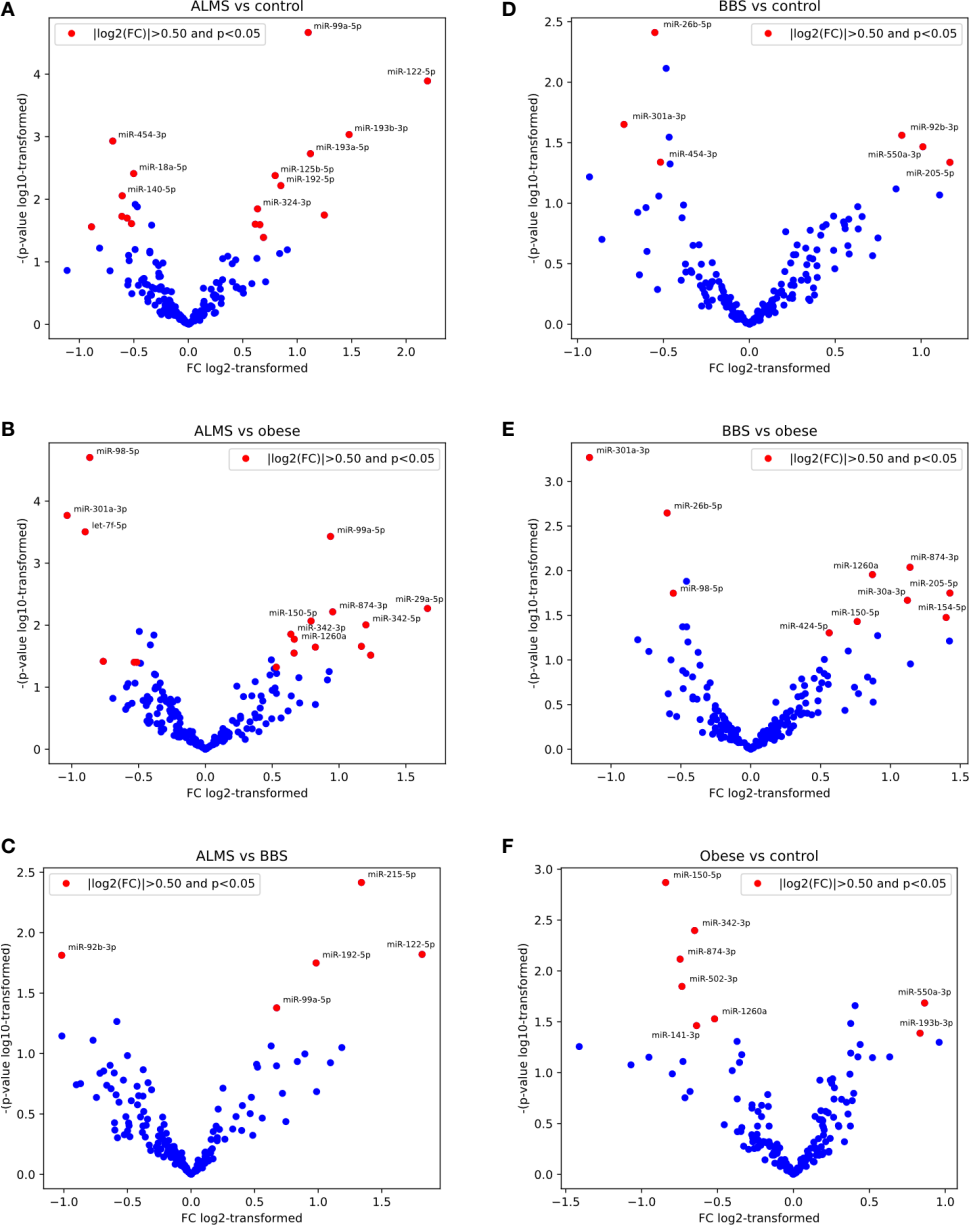
Pairwise results of differential expression analysis for following comparisons: ALMS vs controls **(A)**, obese **(B)** and BBS **(C)**, BBS vs controls **(D)** and obese **(E)**, and obese vs controls **(F)**.

**Figure 3:**
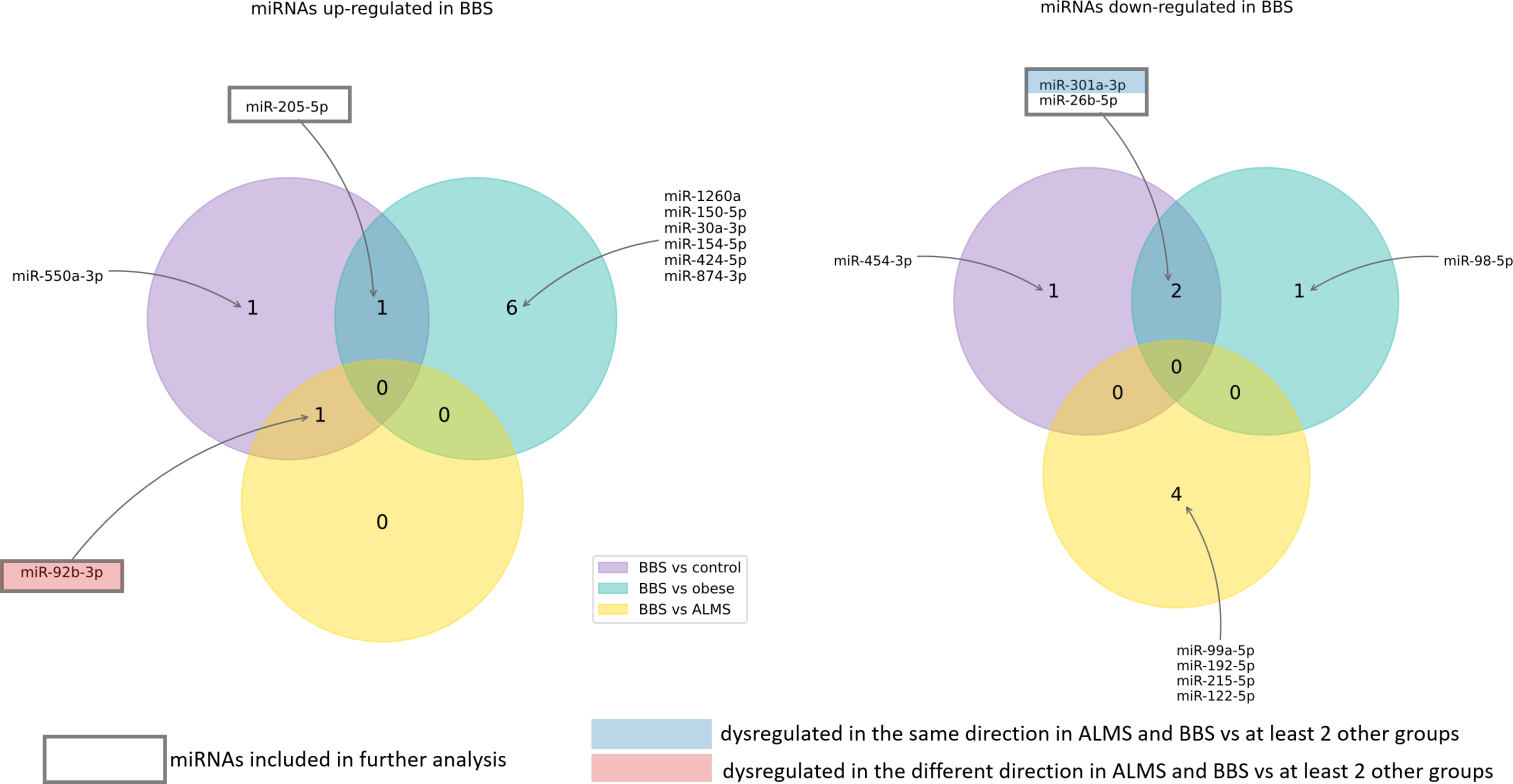
MiRNAs dysregulated in BBS patients.

**Figure 4:**
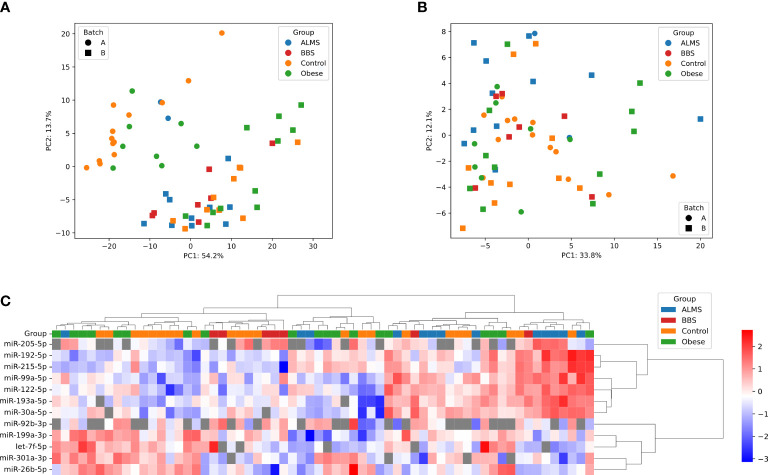
PCA representation of samples before **(A)** and after **(B)** batch effect correction. **(C)** Hierarchical clustering of samples and miRNAs; heatmap shows -ΔCt, scaled per miRNA; distance measure: Euclidean for samples, 1 – correlation coefficient for miRNAs, linkage method: Ward for both dendrograms; undetects are marked with grey.

**Figure 5:**
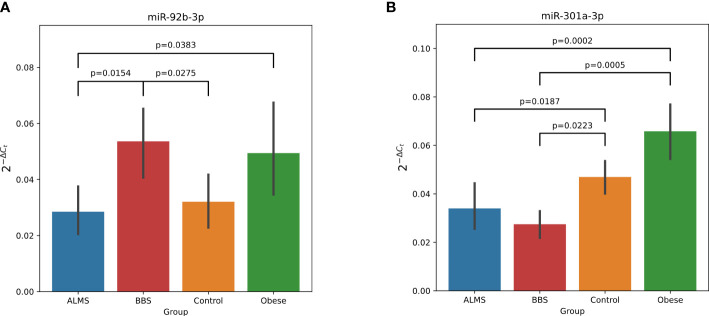
Dysregulation of miR-92b-3p **(A)** and miR-301a-3p **(B)** in ALMS and BBS patients. All statistically significant (p<0.05) differences showed in plots. Y axis represents 2^-ΔCt^.

Among the 178 miRNAs identified after filtering, removing the batch effect and normalization of miRNA expression levels, 19 were detected as up-regulated and 10 down-regulated in ALMS (**Figures 1**, **2A–C**), while 9 up-regulated and 8 down-regulated were found in BBS (**Figures 2C–E**, **3**). Comparing the samples between the four groups, PCA (**Figures 4A, B**) showed some separation between the groups. These pairwise comparisons identified individual miRNAs differently expressed in patients with ALMS versus patients with BBS and obesity and in controls (**Figures 1**–**3**). Furthermore, this allowed the identification of 12 miRNAs dysregulated in BBS or ALMS versus at least two other groups that were included for further analysis. Hierarchical clustering performed with these miRNAs showed some separation between them (**Figure 4C**). Interestingly, 1 miRNA (miR-301a-3p) dysregulated in the same direction in ALMS and BBS relative to at least 2 other groups and 1 miRNA (miR-92b-3p) dysregulated in a different direction in ALMS and BBS patients but relative to at least 2 other groups were selected (**Figure 5**).

**Figure 6:**
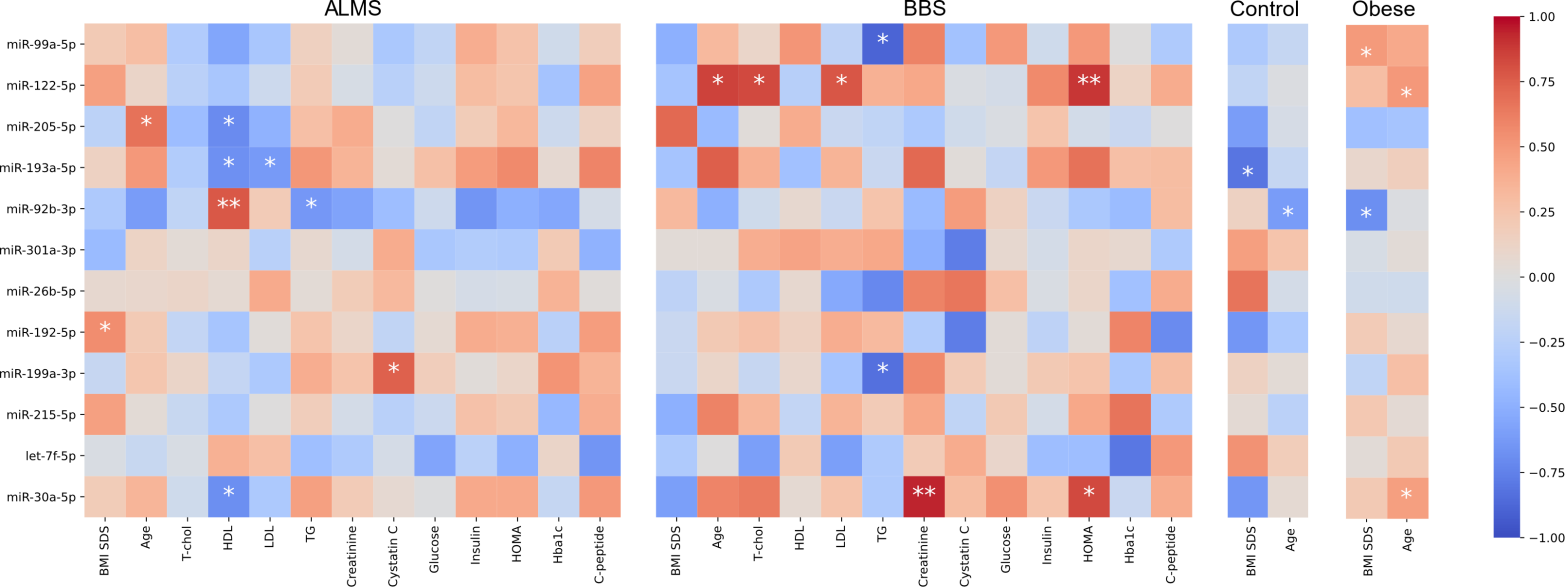
Correlations between miRNA expression (-ΔC_t_) and clinical characteristics. Color represents value of Spearman correlation coefficient, * marks correlations with p<0.05 and ** p<0.01.

We then assessed the potential of these differentially expressed miRNAs as biomarkers of the clinical course of both syndromes. We looked for significant correlations between the levels of the 12 miRNAs, and laboratory results, BMI-SDS and age of patients with ALMS and BBS (**Figure 6**).

Based on this analysis, we found that miR-92b-3p levels were strongly correlated with HDL (R=0.78, p=0.0080) and inversely with TG in ALMS (R=-0.64, p=0.008), but were also inversely correlated with BMI-SDS in obese patients (R=-0.68, p=0.0289) and with age in controls (R=-0.62, p=0.0332). We observed also an inverse relation between levels of miR-193a-5p and both LDL (R=-0.62, p=0.0426) and HDL in ALMS (R=-0.67, p=0.0229), but also with BMI-SDS in controls (R=-0.81, p=0.0149). Moreover, in ALMS, miR-205-5p levels correlated inversely with HDL (R=-0.70, p=0.0347), but also positively with age of patients (R=0.67, p=0.0330). In ALMS, miR-30a-5p levels also correlated negatively with HDL levels (R=-0.68, p=0.0204), while in BBS correlated positively with both HOMA and creatinine levels (R=0.83, p=0.0416 and R=0.94, p=0.0048, respectively), as well as with age in obese patients (R=0.47, p=0.0413). Interestingly, miR-122-5p levels correlated positively in BBS with both total cholesterol (R=0.83, p=0.0212) and LDL (R=0.79, p=0.0362), HOMA values (R=0.89, p=0.0068) and patients’ age (R=0.86, p=0.0137). However, a positive association with age was also observed in obese patients (R=0.51, p=0.0267).

In BBS, miR-99a-5p expression correlated negatively with TG level (R=-0.89, p=0.0188), whereas in obese patients, this expression correlated positively with BMI-SDS (R=0.50, p=0.0353). Furthermore, an inverse correlation was observed between TG and miR-199a-3p levels in BBS (R=-0.83, p=0.0416), while in ALMS, miR-199a-3p expression correlated positively with cystatin C (R=0.75, p=0.0133). A positive correlation was also found between miR-192-5p levels and BMI-SDS in ALMS (R=0.56, p=0.0463).

## Discussion

This study presents the first evaluation of the profile of circulating microRNAs in the serum of patients with ALMS and BBS syndromes, highlighting those that are expressed differentially from those observed in obese patients and healthy individuals. In addition, we identify 8 miRNAs (miR-30a-5p, miR-92b-3p, miR-99a-5p, miR-122-5p, miR-192-5p, miR-193a-5p, miR-199a-3p and miR-205-5p) that show an association with parameters of the clinical course of the syndromes and/or the age of the patients.

Of particular interest are miR-301a-3p, which is down-regulated in both ALMS and BBS relative to obese patients and controls, and miR-92b-3p down-regulated in ALMS and up-regulated in BBS patients, but still significantly different from the other 2 groups. Studies by other authors have shown that miR-301a-3p expression is closely linked to the process of liver fibrosis, making it a promising diagnostic and prognostic marker for the treatment of this pathology (24). In these studies, miRNA-301a-3p expression was upregulated in the progression of liver fibrosis and downregulated in the regression of this process. It appears that miRNA-301a-3p may promote hepatic fibrogenesis and increase the expression of fibrogenic factors, while in regression of liver fibrosis it may induce activation of hepatic stellate cells (HSC) and trigger inflammation (24). It may also be important when considering clinical trials to apply the anti-fibrinolytic properties of the PBI-4050 agent in patients with ALMS (25).

Furthermore, miR-92b-3p, was up-regulated in BBS patients in our study and down-regulated in ALMS, similar to the results of Butler et al, where they related the expression of miRNAs in lymphoblastic cells of obese men with ALMS to their levels in lean men (26). In our study, we showed an inverse correlation between miR-92b-3p and BMI of obese patients and with TG and HDL levels in those with ALMS, which was also observed in patients with inflammatory joint disease (27). In addition, Butler et al. found that miR-30c is up-regulated in ALMS patients (26), while in our study another miRNA from the same family, miR30a-5p, was also up-regulated in ALMS patients and correlated negatively with HDL levels in these patients and positively with HOMA and creatinine levels in BBS patients. Interestingly, in previous studies by other authors, miR-30a-5p has been selected as a biomarker of human kidney disease (28) and risk of developing T2DM (29, 30). Moreover, in the present study, miR-199a-3p levels correlated positively with cystatin C levels in ALMS and negatively with TG levels in BBS patients, similar to the miR-99a-5p expression. Earlier studies by other authors identified both of these miRNAs as being involved in adipogenesis and adipocyte differentiation, particularly in the presence of hyperglycaemia (31, 32). It is also known that overexpression of miR-199a-3p can promote adipocyte proliferation in an animal model by regulating the expression of cell cycle regulatory factors (32), while on the other hand, some authors proposed the use of miR-99a-5p in the treatment of atherosclerosis (33).

In our study, miR-192-5p levels correlated positively with BMI values in ALMS, while miR-193a-5p correlated negatively with LDL and HDL levels in these patients and with BMI in controls. It is worth mentioning that in several studies both of these miRNAs are indicated as potential biomarkers of fatty liver disease (FLD) (34, 35). In addition, a Rotterdam study searching at the population level for circulatory miRNAs as FLD markers indicated that miR-193a-5p could serve as a marker for both FLD and liver fibrosis, similar to miR-122-5p (34). In our study, miR-122-5p showed an association not only with the lipid profile, but also with HOMA as a marker of insulin resistance and the age of patients with BBS. It should be noted that some recent studies by other authors also indicate the involvement of this miRNA in the pathogenesis of childhood obesity (36).

One of the promising markers of clinical course in ALMS, the level of which correlated positively with patient age and negatively with HDL levels in our research, is miR-205-5p. Some literature reports on miR-205-5p in an animal model highlight its role as a modulator of insulin sensitivity (37). Interestingly, other data also indicate that miR-205-5p is among several miRNAs whose serum levels in patients changed as early as 3 weeks after bariatric surgery, showing its role in the pathogenesis of obesity, insulin resistance and T2DM (38).

The dysfunction of the primary ciliary function in ALMS and BBS syndromes involving a number of transcription factors and leading to the development of a range of clinical manifestations, including metabolic disorders in the form of obesity, insulin resistance, lipid abnormalities and type 2 diabetes, results in specific abnormalities in miRNAs expression, which can be observed in their serum levels in ALMS and BBS patients. Therefore, the hypothesis of assessing miRNAs expression in relation to clinical events occurring in patients seems to be very useful in the search for prognostic markers in these syndromes. In particular, as there is a lack of evident genotype-phenotype correlations in both diseases. Moreover, functionally, miRNAs are a biological mechanism aimed at suppressing the expression of unwanted proteins, so the expression of such miRNAs in the serum of patients with ALMS and BBS may provide directions for the development of potential therapeutic interventions.

There are some limitations to our study. First, a severely limited sample of patients with ALMS and BBS was studied due to the extremely low prevalence of these syndromes in the European population (~0.7-1/100,000). In addition, no further validation of the results obtained in the patient validation group was performed. Laboratory tests were not performed in the reference group of obese patients and controls. Due to the limitations of the study, our observations are preliminary at this time. Nevertheless, given the uniqueness of the ALMS and BBS groups and the urgent clinical need for biomarkers of progression of these diseases that would be cheap and available for repeated testing, makes this study a valuable platform for further investigation of miRNAs in longitudinal studies as well as potential therapeutic targets.

In conclusion, our results show for the first time that several selected miRNAs can be used in ALMS and BBS patients as promising markers of the clinical course of the diseases. However, further studies are needed to clearly establish the association of changes in miRNA expression with disease progression and to validate the results to assess the potential use of these markers in the future.

## Data availability statement

The data presented in the study are deposited in the GEO repository, found online at https://www.ncbi.nlm.nih.gov/geo/query/acc.cgi?, accession number GSE214735.

## Ethics statement

The studies involving human participants were reviewed and approved by The study protocol was approved by the University Bioethics Committee at the Medical University in Lodz, Poland (RNN/343/17/KE). Written informed consent to participate in this study was provided by the participants’ legal guardian/next of kin.

## Author contributions

AZ collected clinical data and wrote the draft of the manuscript. US and MS performed statistical analyses. KJ and AS. collected clinical data. WF performed statistical analyses and designed the study. MB performed genetic analyses. All authors contributed to the article and approved the submitted version.

## Funding

This study was supported by National Science Centre grant No 2018/29/B/NZ5/00330. AZ and WF are the guarantors of this work and, as such, had full access to all the data in the study and take responsibility for the integrity of the data and the accuracy of the data analysis.

## Conflict of interest

The authors declare that the research was conducted in the absence of any commercial or financial relationships that could be construed as a potential conflict of interest.

## Publisher’s note

All claims expressed in this article are solely those of the authors and do not necessarily represent those of their affiliated organizations, or those of the publisher, the editors and the reviewers. Any product that may be evaluated in this article, or claim that may be made by its manufacturer, is not guaranteed or endorsed by the publisher.

## Supplementary material

Supplementary Data Sheet 1 Raw C_q_ values of miRNA expression (only background-filtered).

Supplementary Data Sheet 2 MiRNA expression after filtration (-ΔC_t_), batch effect removal and normalization.

Supplementary Data Sheet 3 Results of differential expression analysis in limma for all pairwise comparisons between groups.
